# Study of atopic multimorbidity in subjects with rhinitis using multiplex allergen component analysis

**DOI:** 10.1186/s13601-020-0311-6

**Published:** 2020-02-21

**Authors:** Viiu Blöndal, Fredrik Sundbom, Magnus P. Borres, Marieann Högman, Kjell Alving, Andrei Malinovschi, Christer Janson

**Affiliations:** 1 Medical Sciences, Respiratory, Allergy and Sleep Research, Uppsala University, Uppsala, Sweden; 2 Women’s and Children’s Health, Uppsala University, Uppsala, Sweden; 3 Thermo Fischer Scientific, Uppsala, Sweden; 4 Department of Medical Sciences, Lung Allergy and Sleep Research, Uppsala University Hospital, Uppsala University, 751 85 Uppsala, Sweden

**Keywords:** Rhinitis, Asthma, Eczema, Atopic multimorbidity, Multiplex component analysis

## Abstract

**Background:**

Rhinitis is a common problem within the population. Many subjects with rhinitis also have atopic multimorbidity, such as asthma and eczema. The purpose of this investigation was to compare subjects with only rhinitis to those that have rhinitis, asthma and/or eczema in relation to immunoglobulin E (IgE) sensitization, inflammatory markers, family history, lung function and body mass index (BMI).

**Methods:**

A total of 216 adult subjects with rhinitis from the European Community Respiratory Health Survey II were investigated with multiplex component allergen analysis (103 allergen components), total IgE, C-reactive protein, eosinophilic cationic protein, fractional exhaled nitric oxide and spirometry. Rhinitis, eczema, asthma and parental allergy were questionnaire-assessed.

**Results:**

Of the 216 participants with rhinitis, 89 also had asthma and/or eczema. Participants with rhinitis that also had asthma or eczema were more likely to be IgE-sensitized (3.44, odds ratio, OR: 95% CI 1.62–7.30, adjusted for sex, age, mother’s allergy, total IgE and forced expiratory volume (FEV_1_)). The number of IgE-positive components was independently associated with atopic multimorbidity (1.11, OR: 95% Cl 1.01–1.21) adjusted for sex, age, mother’s allergy, total IgE and FEV_1_. When analysing different types of sensitization, the strongest association with atopic multimorbidity was found in participants that were IgE-sensitized both to perennial and seasonal allergens (4.50, OR: 95% CI 1.61–12.5). Maternal allergy (2.75, OR: 95% CI 1.15–4.46), high total IgE (2.38, OR: 95% CI 1.21–4.67) and lower FEV_1_ (0.73, OR: 95% CI 0.58–0.93) were also independently associated with atopic multimorbidity, while no association was found with any of the other inflammatory markers.

**Conclusion:**

IgE polysensitization, to perennial and seasonal allergens, and levels of total IgE seem to be the main determinants of atopic multimorbidity in subjects with rhinitis. This indicates that disease-modifying treatment that targets IgE sensitization may be of value when decreasing the risk of developing atopic multimorbidity.

## Background

Rhinitis is a common problem [1–3] and is troublesome on its own, but it has also been shown to be a risk factor for developing bronchial hyper-responsiveness and asthma [4, 5]. Multimorbidity involving the triad of rhinitis, asthma and eczema exists among both atopic and non-atopic individuals, although the prevalence is higher among atopic individuals [6–9]. About 74–90% of subjects with allergic asthma also have rhinitis [5, 10, 11]. Rhinitis, irrespective of atopic status, is a risk factor for developing asthma, but it has also been found that the co-occurrence of the two conditions is much more likely in atopic individuals [2, 3, 11]. The worldwide prevalence of allergic rhinitis, asthma and eczema in children under 18 years of age is 13%, 12% and 8% respectively [1]. The corresponding prevalence among adults is 4.5% for asthma and 20.9% for rhinitis [12]. Eczema among adults is estimated at 7.1% [13]. The prevalence of having all three atopic disorders was 1% in children [1]. The likelihood of having all three conditions together was 10 times higher than could be expected by chance [1, 14].

Multimorbidity increases the socioeconomic and psychological burden of atopic disorders [15, 16]. Considerable research has focused on discovering the risk factors for each atopic disorder separately, [5–7, 14, 17–19] but the reason why some individuals are limited to just having rhinitis and some develop additional atopic disorders is still not entirely known.

In one study, there was no difference in systemic or local eosinophilic inflammation between subjects with birch pollen allergy who had rhinitis or rhinitis and asthma [9]. There was also no difference in the pattern of degranulation for eosinophils and neutrophils after allergen exposure between these two groups [20]. Aronson et al. found that participants with allergic rhinitis and bronchial hyper-reactivity (BHR) had lower exhaled nitric oxide (F_E_NO) levels than those with allergic rhinitis and asthma [5]. In another study, participants with asthma had higher levels of leukotrienes in induced sputum than those with rhinitis [21]. It has been reported that individuals with rhinitis and asthma have higher peripheral F_E_NO levels than those with only rhinitis and that subjects with allergic rhinitis and asthma have more peripheral airway obstruction after a methacholine challenge than those with only allergic rhinitis [4, 10]. This indicates that peripheral airway inflammation distinguishes those with allergic rhinitis and asthma from those with only allergic rhinitis. According to some authors developing allergic rhinitis is more strongly associated with sensitization to seasonal allergens, [6, 11, 22] whereas those that developed asthma were more likely to be sensitised to perennial allergens, compared with seasonal allergens [11, 22, 23].

The multiplex allergen component technique has significantly increased our understanding of atopic multimorbidity, allowing for a more detailed insight into the profiles of atopic individuals. This technique has several advantages compared with extract-based tests. Breaking the IgE-mediated response into components allows us to see patterns of IgE sensitisation to various proteins which are associated with different clinical symptoms [24].

The aim of this investigation was to compare subjects with only rhinitis with those that have rhinitis in combination with asthma and/or eczema in relation to IgE sensitisation, inflammatory markers, family history, lung function and BMI.

## Methods

### Population

**Fig. 1:**
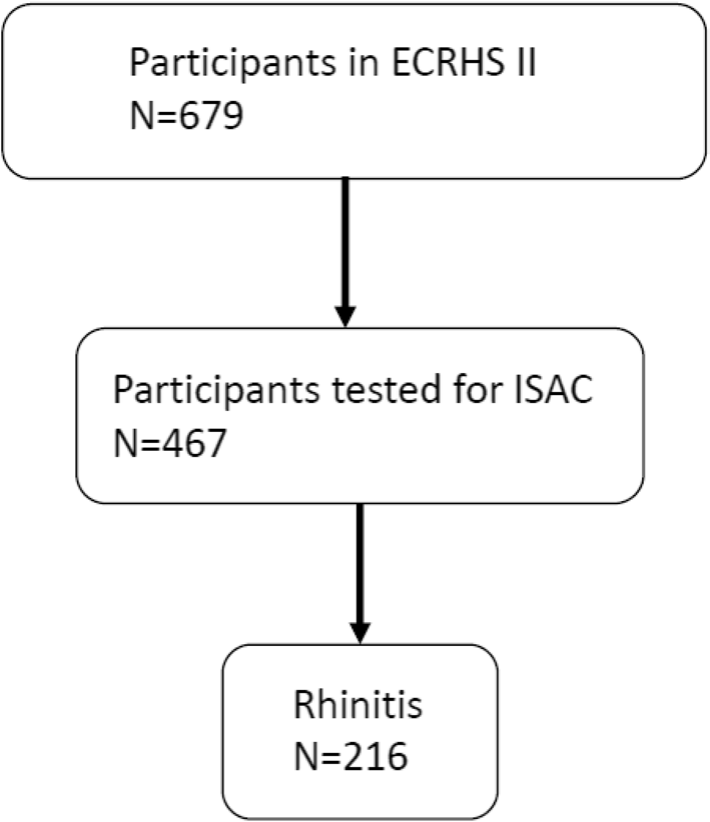
Flow chart for the inclusion of subjects

The study was based on subjects who participated in the European Community Respiratory Health Survey (ECRHS II), which is the 10-year follow-up of an international multicentre study of asthma and allergy. The design of ECRHS I and II has been published in detail [25]. Subjects who had participated in Stage 2 of ECRHS I were invited to participate in ECRHS II. Each participant was sent a brief questionnaire (Stage 1) and, among those who responded, a random sample was invited to undergo a more detailed clinical examination (Stage 2). A ‘‘symptomatic’’ sample consisting of additional subjects who reported symptoms of waking with shortness of breath, asthma attacks, or using asthma medication in Stage 1 was also studied. In ECRHS II, 679 subjects from ECRHS I were re-investigated. The subjects answered a standardised questionnaire administered by trained interviewers. A total of 467 subjects were examined with ImmunoCAP ISAC and underwent lung function tests and blood tests for analyses of inflammatory markers. This study comprised 216 people with allergic rhinitis who were examined with ImmunoCAP ISAC (Fig. 1). Among the subjects with allergic rhinitis, 70 had asthma.

### Questionnaires

The ECRHS II main questionnaire (http://www.ecrhs.org) [26] was used to obtain information about respiratory symptoms, smoking history, parental allergy and asthma.

Rhinitis was defined as having had problems sneezing, or a runny or blocked nose when not having a cold in the last 12 months [8].

Eczema was defined as having an itchy rash that came and went for at least 6 months and having had this problem within the past 12 months [27].

Asthma was defined as ever being diagnosed with asthma and having had an asthma attack or one of the following symptoms during the past 12 months: nocturnal chest tightness, attack of shortness of breath, chest wheezing, or whistling [28].

### Allergy testing

The presence of IgE antibodies was examined using microarray chip technology (ImmunoCAP ISAC; Thermo Fischer Scientific, Uppsala, Sweden) [29, 30]. The chip had 103 native or recombinant allergen components from 43 allergen sources. Specific IgE was reported as ISAC Standardised Units (ISU), which is a semi-quantitative estimate of the actual specific IgE titre. Subjects were regarded as being non-IgE sensitised if the signal could not be measured or was very low (< 0.3 ISU). The IgE sensitisation pattern was grouped into four allergen sources categories: food allergens, seasonal allergens (pollen), perennial allergens (animal, mite and mould) and other allergen sources (latex, annual mercury, bee venom, anisakis).

### Inflammatory markers

Exhaled nitric oxide (F_E_NO) measurements were made using an exhalation flow of 50 mL/s [31]. The system used for NO measurements was a computer-based single-breath NO system from Nitrograf (Hässelby, Sweden) that used a chemiluminescence analyser (Sievers NOA 280; Sievers, Boulder, Col, USA). Peripheral or alveolar F_E_NO was estimated by calculating the flow-independent alveolar nitric oxide concentration (C_A_NO) was estimated by analysing the fractional exhaled NO concentration obtained at three different rates (5, 100 and 500 mL s^−1^) and the Högman-Meriläinen algorithm [32].

Blood samples were collected for eosinophil cationic protein (ECP). Samples were kept at 24 °C for 60 min before centrifuging. The concentration of s-ECP was assayed with a double antibody radioimmunoassay (Pharmacia diagnostics, Uppsala, Sweden). Blood samples collected for C-reactive protein (CRP) were analysed on a Hitachi 911 analyser using a commercially available latex-enhanced immunoturbidimetric assay from Roche. The lower detection limit of the assay is 0.1 mg/L.

### Lung function

FEV_1_ was measured using a dry rolling-seal spirometer (Model 2130; SensorMedics, Anaheim, Cal, USA). Up to five technically acceptable manoeuvres were measured. American Thoracic Society recommendations were followed [33]. The predicted values for FEV_1_ were calculated on the basis of European Coal and Steel Union reference values [34]. Weight and height were measured and body mass index (BMI) was calculated.

### Statistical methods

Statistical analyses were performed using STATA (14; StataCorp, College Station, Tex). Non-normally distributed variables, F_E_NO, total IgE and ECP, were log transformed before analysis. The material was divided into four groups according to the prevalence of atopic conditions: rhinitis only; rhinitis and eczema; rhinitis and asthma; rhinitis, asthma and eczema. The *χ*^*2*^ test and ANOVA with Bonferroni correction were used when comparing these four groups. Logistic regression was used when analysing an independent association to having rhinitis with asthma and/or eczema as opposed to only having rhinitis. A P value of < 0.05 was considered statistically significant.

## Results

**Fig. 2:**
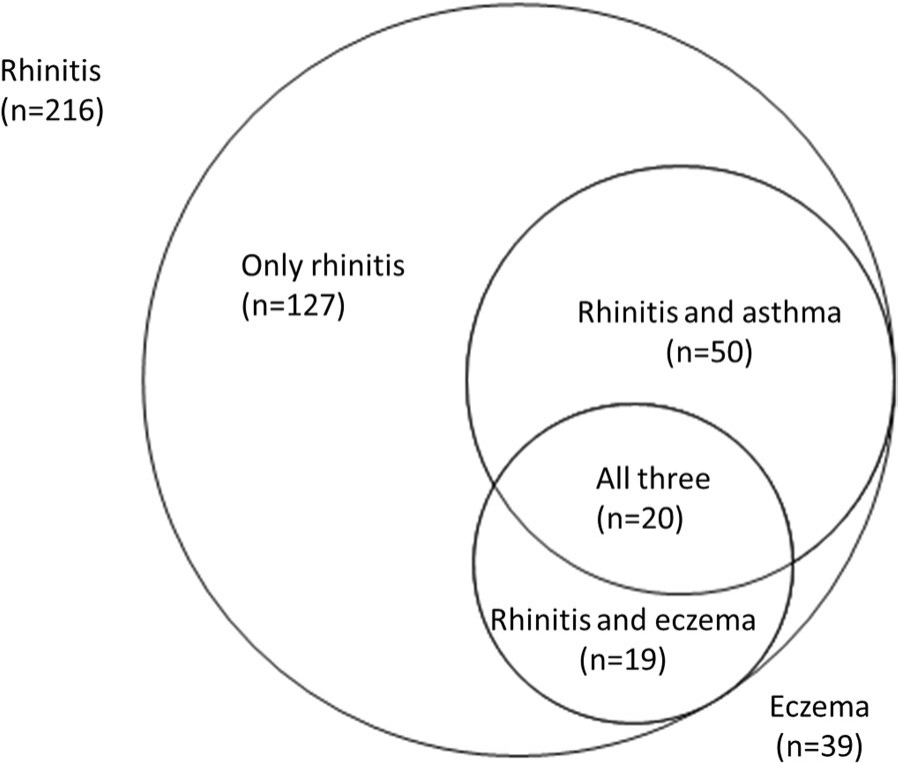
Asthma and eczema in participants with rhinitis. *The distribution of rhinitis subjects (n = 216) and subjects with eczema (n = 39) within different subgroups

This study comprised 216 participants with rhinitis, of whom 89 also had asthma and/or eczema (Fig. 2). The participants were divided into four groups based on the presence or absence of asthma and or eczema. The characteristics of these four groups are presented in Table 1. Significant group differences were found for BMI and FEV_1_, with the lowest mean value for both variables in the group with both asthma and eczema. Looking at individual groups, FEV_1_ was significantly lower in the groups with asthma and the group with asthma and eczema compared with those with only rhinitis (P < 0.001). No difference in FEV1 was found between subjects with rhinitis and those with both rhinitis and eczema. BMI was significantly lower in the group with asthma and eczema compared with those that only had asthma (P = 0.04).

**Table 1:** Characteristics of the participants based on whether they had asthma and or eczema

	Rhinitis (n = 127)	Rhinitis and eczema (n = 19)	Rhinitis and asthma (n = 50)	Rhinitis, asthma, eczema (n = 20)	*P* value
Female gender	47%	68%	56%	40%	0.22
Age (years)	43 ± 7	41 ± 8	41 ± 7	39 ± 7	0.13
BMI (kg/m^2^)	24 ± 4	25 ± 4	26 ± 5	23 ± 3	0.04
Smoking history					0.80
Never smoked	48%	47%	50%	65%	
Ex-smoker	34%	26%	32%	20%	
Current smoker	18%	26%	18%	15%	
Parental heredity					
Mother allergy	27%	58%	48%	45%	0.006
Mother asthma	15%	37%	22%	10%	0.08
Father allergy	28%	21%	26%	40%	0.58
Father asthma	8%	5%	8%	19%	0.96
FEV_1_ (% of predicted)	107 ± 13	111 ± 11	98 ± 13	93 ± 15	< 0.001
Medication in the last 12 months					
Nasal steroids	17%	21%	30%	32%	0.17
Antihistamines	30%	47%	50%	70%	0.002
Inhaled corticosteroids	9%	5%	48%	56%	< 0.001

Data presented as % and mean ± SD

There was a significant group difference in relation to having an allergic mother, with the higher prevalence in the group with rhinitis and eczema. No other significant association was found for parental allergy or asthma. There was also a significant difference in relation to medication, with the highest use of antihistamines and inhaled corticosteroids in the group that had all three atopic disorders (Table 1).

### Inflammatory markers and total IgE

There was a significant difference between the groups regarding total IgE but not in relation to other inflammatory markers (Table 2). Looking at individual groups, total IgE was significantly higher in the groups with rhinitis and asthma (P < 0.001) and the group with rhinitis, asthma and eczema compared with those with only rhinitis (P < 0.001).

**Table 2:** Inflammatory markers and total IgE in the participants (geometric mean (95% confidence interval)

	Rhinitis (n = 127)	Rhinitis and eczema (n = 19)	Rhinitis and asthma (n = 50)	Rhinitis, asthma, eczema (n = 20)	P-value
F_E_NO^a^ ppb	20 (17–23)	23 (14–35)	27 (20–37)	22 (15–33)	0.26
C_A_NO^a^ ppb	1.3 (1.1–1.6)	1.3 (0.8–2.2)	1.2 (0.8–1.7)	1.9 (1.0–3.5)	0.56
ECP^b^ (µg/L)	8.0 (7.0–9.1)	6.8 (4.9–9.5)	9.6 (8.1–11.4)	8.9 (6.7–11.9)	0.19
CRP (mg/L)	1.0 (0.9–1.3)	1.3 (0.73–2.2)	1.2 (0.8–1.6)	0.7 (0.4–1.3)	0.45
Total IgE (kU/L)	43 (35–54)	44 (26–77)	112 (78–156)	191 (106–342)	< 0.0001

*F*_*E*_*NO* exhaled nitric oxide, *C*_*A*_*NO* the estimated alveolar NO concentration, *ECP* eosinophil cationic protein, *CRP* C-reactive protein

^a^Available in 128 participants

^b^Available in 178 participants

### IgE sensitisation

**Fig. 3:**
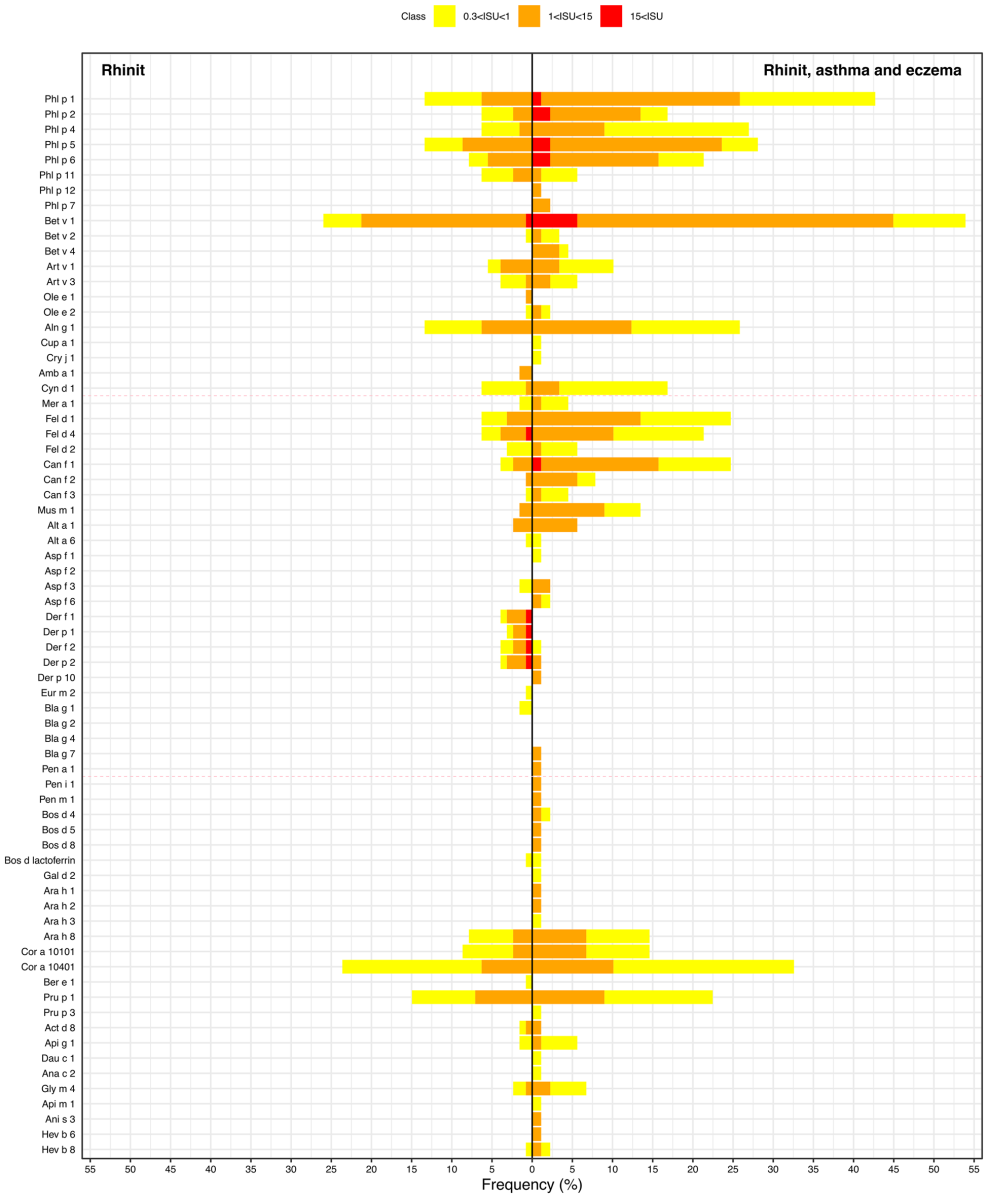
Sensitisation to allergen components in the ISAC panel. *The column on the left represents subjects with only rhinitis and the column on the right subjects with rhinitis that also have eczema, asthma or both. The darker the colour (yellow, orange, red), the higher the test result

**Fig. 4:**
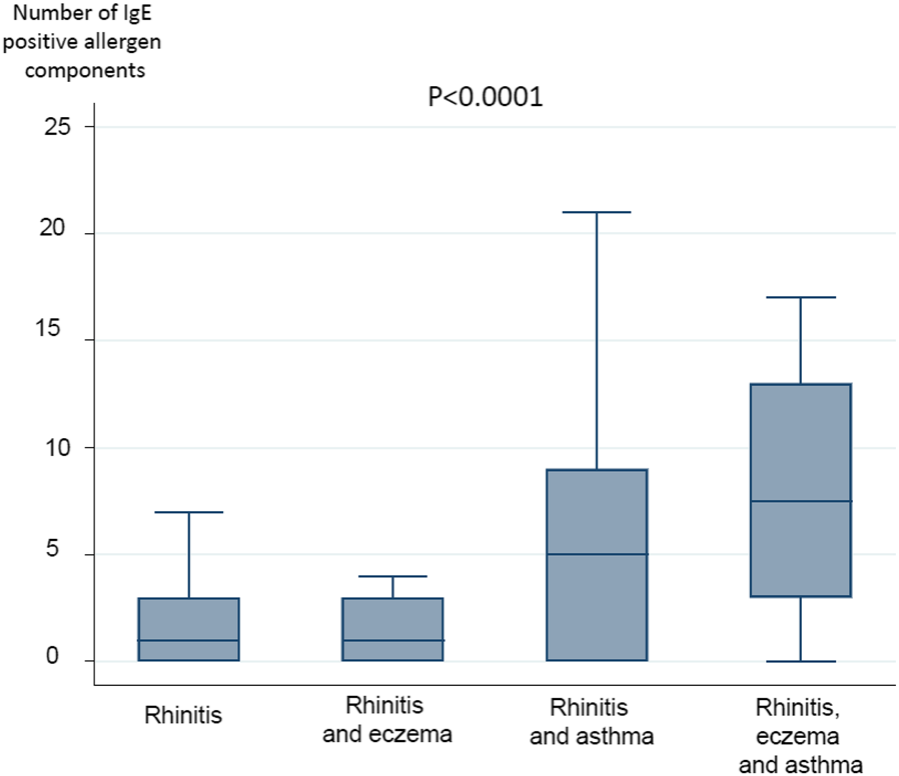
Association between the number of IgE-positive allergen components and having rhinitis with asthma/eczema. *median and interquartile range

IgE sensitisation to the different allergen components in the ISAC panel is presented in Fig. 3 and Additional file 1. There were significant group differences for many types of IgE sensitisation, with the highest prevalence in the group with rhinitis, eczema and asthma (Table 3). There were also significant group differences in the number of IgE-positive allergen components, with the highest number in the group with rhinitis, eczema and asthma (Fig. 4). In addition, the sensitization pattern between the rhinitis participants and participants with rhinitis, eczema and asthma differs in terms of IgE levels (Fig. 3).

**Table 3:** IgE sensitisation in the participants

	Rhinitis (n = 127)	Rhinitis and eczema (n = 19)	Rhinitis and asthma (n = 50)	Rhinitis, asthma, eczema (n = 20)	P-value
Grass pollen	20%	21%	56%	65%	< 0.001
Tree pollen	26%	32%	56%	75%	< 0.001
Food of plant origin	29%	32%	42%	65%	0.01
Furry animals	11%	21%	44%	55%	< 0.001
Moulds	5%	0%	8%	20%	0.04
Mites	5.5%	0%	2%	0%	0.10
Seasonal	38%	37%	64%	75%	0.001
Perennial	17%	21%	50%	60%	< 0.001
Food	30%	32%	44%	65%	0.01
Any sensitisation	41%	58%	80%	83%	< 0.001

Seasonal: grass and tree pollen; Perennial: furry animal, mite and mould

### Multivariable analyses

Mother’s allergy, high total IgE, low FEV_1_, being IgE sensitised and the number of IgE-positive allergen components were independently associated with having rhinitis with asthma and or eczema (Table 4). When analysing different types of sensitisation, the strongest association was found in participants that were IgE sensitised to both perennial and seasonal allergens. Of the perennial allergens only sensitization to furry animals and moulds had a significant association to atopic multimorbidity (Table 3). The association between total IgE and atopic multimorbidity remained significant after adjusting for IgE sensitization to specific allergens [adjusted OR (95% CI) 2.38 (1.21–4.69)].

**Table 4:** Variables associated with having asthma with eczema and/or asthma compared with only having rhinitis

	Crude OR (95% CI)	Adjusted OR^a^ (95% CI)
Mother’s allergy	2.67 (1.50–4.73)	2.29 (1.16–4.55)
Total IgE (log increase)	3.63 (2.08–6.36)	2.51 (1.25–5.03)
FEV_1_ per 10% of predicted increase	0.02 (0.00–0.16)	0.03 (0.00–0.38)
Number of IgE-positive allergen components	1.15 (1.08–1.23)	1.11 (1.01–1.21)
Seasonal	2.5 (1.45–4.42)	2.03 (0.96–4.27)
Perennial	4.31 (2.30–8.06)	2.43 (1.10–5.36)
Food	2.00 (1.13–3.51)	1.35 (0.67–2.71)
Any sensitisation	4.41 (2.41–8.09)	3.20 (1.49–6.86)
No sensitisation		1
Seasonal but not perennial	1.39 (0.68–2.81)	1.19 (0.50–2.86)
Perennial but not seasonal	2.13 (0.66–6.87)	0.74 (0.19–2.95)
Seasonal and perennial	6.21 (2.92–13.21)	4.36 (1.54–12.35)

*(OR (95% CI)* odds ratio (95% CI)

^a^Adjusted for gender, age, mother’s allergy, total IgE, BMI and FEV_1_

## Discussion

The main finding in the present study is that participants with allergic rhinitis that also had asthma and/or eczema were more likely to have IgE polysensitisation, higher total IgE, a history of maternal allergy and lower lung function than subjects that only had rhinitis.

We have used a multiplex allergen component technique to evaluate the association between atopic multimorbidity and IgE sensitisation. Subjects with only allergic rhinitis were sensitized to fewer allergens, compared with those with allergic rhinitis, asthma and eczema. This is in accordance with previous observations from other studies [8, 11]. Sensitisation to more allergens is associated with more severe forms of asthma, eczema and allergic rhinitis individually [18, 19, 24, 35–37]. Participants with all three disorders were more likely to be sensitised to perennial allergens. The risk was even higher if the participants were sensitised to both perennial and seasonal allergens. This is in accordance with studies showing that polysensitisation is common in individuals that have both asthma and rhinitis compared with those that only have rhinitis [6, 11, 22]. Previous studies have shown that participants with eczema also display a greater tendency towards polysensitisation [6]. We also found that high total IgE is associated with atopic multimorbidity, even after adjusting for specific IgE sensitisation. This is in accordance with previous findings [38, 39].

Having an allergic mother was more common in participants with rhinitis and concomitant asthma and eczema, whereas no significant association was found for paternal allergy or asthma. Previous studies have shown that subjects with a maternal history of allergies run a four times higher risk, whereas paternal allergy was associated with a two times higher risk of having two or more atopic disorders [11, 40].

No statistically significant difference was found for any of the inflammatory markers in this study when comparing those with atopic multimorbidity with those with only rhinitis. Previous studies have found higher F_E_NO levels in participants with both allergic rhinitis and asthma as opposed to just allergic rhinitis [5, 10]. Levels of F_E_NO have a quantitative relationship with the degree of IgE sensitisation and are associated with a higher degree of bronchial responsiveness, obstruction and risk of developing asthma [41, 42]. Previous studies have indicated that a more peripheral airway inflammation distinguishes those with asthma and rhinitis from those with only rhinitis [4, 5]. There was, however, no association between the flow-independent alveolar NO concentration and having atopic multimorbidity in the present study. It is possible to speculate that the F_E_NO values may have been influenced by the higher use of inhaled corticosteroids that was observed with increasing atopic multimorbidity [43]. We did not find a significant difference in serum ECP levels between the groups, suggesting that there is no difference in eosinophilic inflammation. This is in accordance with other studies showing the same degree of eosinophilic inflammation in blood, nasal mucosa and bronchial mucosa during the pollen season, in subjects with only rhinitis and those with rhinitis in combination with asthma [9, 20, 21, 44]. We did not find any association between atopic multimorbidity and CRP and this is in accordance with the results from previous findings [28].

In the present study, FEV_1_ was significantly lower in the groups with asthma and the group with asthma and eczema compared with those with only rhinitis.

The strength of these data is that the study is population based. An extensive IgE analysis was performed using the multiplex allergen component technique which minimises the risk of missing relevant allergens. One limitation is that the group categorisation was based on self-reported data and the relatively small sample size could affect the statistical results. Furthermore, inflammatory markers were not available for all subjects. The definition of eczema in our study could also cover other skin conditions and lead to an overestimation of the prevalence of eczema in our study.

Evaluating a polysensitised patient in clinical practice is challenging. The multiplex component-based allergen microarray has great potential for use both clinically and in research. It has been shown that IgE sensitisation to grass pollen precedes allergic symptoms by several years, [24] starting with a mono- or oligosensitisation to allergen components during the preclinical and early stages of allergic rhinitis [24]. In future, testing at-risk individuals could be used to find patients that might benefit from specific allergen immunotherapy to prevent more severe atopic multimorbidity later in life [45–47].

## Conclusion

IgE polysensitization, namely to perennial and seasonal allergens, and levels of total IgE appear to be one of the main differences between subjects with atopic multimorbidity compared with those with only rhinitis. This indicates that disease-modifying treatment targeting IgE sensitisation, such as specific allergen immunotherapy, may be of value in reducing the risk of developing atopic multimorbidity. More prospective studies that include adult participants are warranted in order to investigate this further.

## Supplementary information

**Additional file 1.** Sensitisation to allergen components in the ISAC panel in %.

## Data Availability

The dataset is held and managed by the Department of Medical Sciences, Uppsala University, Uppsala, Sweden. Data cannot be made freely available as they are subject to secrecy in accordance with the Swedish Public Access to Information and Secrecy Act, but can be made available to researchers upon request (subject to a review of secrecy). Requests for data can be sent to the Unit for Respiratory, Allergy and Sleep Research at the University Hospital in Uppsala lungforskning@akademiska.se.

## Abbreviations

BMI: Body mass index
C_A_NO: Alveolar nitric oxide concentration
CRP: C-reactive protein
ECP: Eosinophilic cationic protein
ECRHS: European Community Respiratory Health Survey
F_E_NO: Fractional exhaled nitric oxide
FEV1: Forced expiratory volume in 1 s
IgE: Immunoglobulin E
