# Supplementary material for: Study of atopic multimorbidity in subjects with rhinitis using multiplex allergen component analysis
Source: Clin Transl Allergy. 2020 Feb 21;10:6. doi: 10.1186/s13601-020-0311-6 (PMC7033937; doi:10.1186/s13601-020-0311-6)
Supplement: Supplementary file 1 — Additional file 1. Sensitisation to allergen components in the ISAC panel in %. [file 13601_2020_311_MOESM1_ESM.pdf]

Additional file 1

Sensitisation to allergen components in the ISAC panel in %

Allergen Component	Allergen source	Rhinitis (n=127)	Rhinitiz and eczema (n=19)	Rhinitis and asthma (n=50)	Rhinitis, asthma, eczema (n=20)
Phl p 1	Timothy	13.4	15.8	48.0	55.0
Phl p 2	Timothy	7.1	5.3	20.0	20.0
Phl p 4	Timothy	6.3	15.8	30.0	30.0
Phl p 5	Timothy	13.4	10.5	34.0	30.0
Phl p 6	Timothy	7.9	5.3	28.0	20.0
Phl p 11	Timothy	6.3	5.3	6.0	10.0
Phl p 12	Timothy	0.0	0.0	0.0	5.0
Phl p 7	Timothy	0.0	0.0	4.0	0.0
Bet v 1	Birch	26.0	31.6	56.0	70.0
Bet v 2	Birch	0.8	0.0	0.0	15.0
Bet v 4	Birch	0.0	0.0	6.0	5.0
Art v 1	Mugwort	5.5	5.3	12.0	10.0
Art v 3	Mugwort	4.0	5.3	8.0	0.0
Ole e 1	Olive	0.8	0.0	0.0	0.0
Ole e 2	Olive	0.0	0.0	0.0	10.0
Pla l 1	Plantain	0.0	0.0	0.0	0.0
Aln g 1	Alder	13.4	5.3	26.0	45.0
Cup a 1	Cypress	0.0	0.0	2.0	0.0
Cry j 1	Japanese cedar	0.0	0.0	2.0	0.0
Amb a 1	Rag weed	1.6	0.0	0.0	0.0
Cyn d 1	Bermuda grass	6.3	10.5	18.0	20.0
Mer a 1	Annual mercury	1.6	0.0	2.0	15.0
Fel d 1	Cat	6.3	10.5	26.0	35.0
Fel d 4	Cat	6.3	0.0	28.0	25.0
Fel d 2	Cat	3.2	5.3	4.0	10.0
Can f 1	Dog	4.0	10.5	24.0	40.0
Can f 2	Dog	0.8	0.0	6.0	20.0
Can f 3	Dog	0.8	5.3	4.0	5.0
Mus m 1	Mouse	1.6	0.0	18.0	15.0
Alt a 1	Alternaria	2.4	0.0	4.0	15.0
Alt a 6	Alternaria	0.8	0.0	0.0	5.0
Asp f 1	Aspergillus	0.0	0.0	2.0	0.0
Asp f 2	Aspergillus	0.0	0.0	0.0	0.0
Asp f 3	Aspergillus	1.6	0.0	2.0	5.0
Asp f 6	Aspergillus	0.0	0.0	2.0	0.5
Der f 1	House dust mite	3.94	0.0	0.0	0.0

Der p 1	House dust mite	3.2	0.0	0.0	0.0
Der f 2	House dust mite	3.9	0.0	2.0	0.0
Der p 2	House dust mite	3.9	0.0	2.0	0.0
Der p 10	House dust mite	0.0	0.0	2.0	0.0
Eur m 2	House dust mite	0.8	0.0	0.0	0.0
Bla g 1	Cockroach	1.6	0.0	0.0	0.0
Bla g 2	Cockroach	0.0	0.0	0.0	0.0
Bla g 4	Cockroach	0.0	0.0	0.0	0.0
Bla g 7	Cockroach	0.0	0.0	2.0	0.0
Pen a 1	Shrimp, brown	0.0	0.0	2.0	0.0
Pen i 1	Shrimp, indian	0.0	0.0	2.0	0.0
Pen m 1	Shrimp, black tiger	0.0	0.0	2.0	0.0
Bos d 4	Milk	0.0	0.0	2.0	5.0
Bos d 5	Milk	0.0	0.0	0.0	5.0
Bos d 8	Milk	0.0	0.0	0.0	0.5
Bos d lactoferrin	Milk	0.8	0.0	0.0	5.0
Gal d 2	Egg white	0.0	0.0	0.0	5.0
Ara h 1	Peanut	0.0	0.0	0.0	5.0
Ara h 2	Peanut	0.0	0.0	0.0	5.0
Ara h 3	Peanut	0.0	0.0	0.0	5.0
Ara h 8	Peanut	7.9	5.3	20.0	10.0
Cor a 10101	Hazel nut	8.7	0.0	18.0	20.0
Cor a 10401	Hazel nut	23.6	31.6	30.0	40.0
Ber e 1	Brazil nut	0.8	0.0	0.0	0.0
Aal d 1	Apple	14.2	10.5	31.0	45.0
Pru p 1	Peach	15.0	10.5	22.0	35.0
Pru p 3	Peach	0.0	0.0	2.0	0.0
Act d 8	Kiwi	1.6	0.0	2.0	0.0
Api g 1	Celery	1.6	0.0	4.0	15.0
Dau c 1	Carrot	0.0	0.0	2.0	0.0
Ana c 2	Bromelain, Pineapple	0.0	0.0	2.0	0.0
Gly m 4	Soybean	2.4	0.0	12.0	0.0
Api m 1	Bee venom	0.0	5.3	0.0	0.0
Ani s 3	Anisakis	0.0	0.0	2.0	0.0
Hev b 6	Latex	0.0	0.0	0.0	5.0
Hev b 8	Latex	0.8	0.0	0.0	10.0
